# A TEMPO-loaded DNA hydrogel enabling integrated early diagnosis and treatment of osteoarthritis

**DOI:** 10.1186/s12951-025-03794-0

**Published:** 2025-10-31

**Authors:** Hong Huang, Mingze Tang, Pengcheng Hu, Yingshi Zhan, Wei Sun, Weipeng Zheng, Jianwei Zhu, Jianmao Chen, Song Xue, Shiqian Huang, Weiyu Han, Chao Zhang, Changhai Ding, Yan Zhang, Shushu Li, Guangfeng Ruan

**Affiliations:** 1 Department of Orthopedics, Guangzhou First People’s Hospital, School of Medicine, South China University of Technology, Guangzhou, 510180 Guangdong China; 2 Clinical Research Centre, Guangzhou First People’s Hospital, Guangzhou Medical University, Guangzhou, 510180 Guangdong China; 3 Clinical Research Center, Zhujiang Hospital, Southern Medical University, Guangzhou, 510282 Guangdong China; 4 Centre of Orthopedics, Zhujiang Hospital, Southern Medical University, Guangzhou, 510282 Guangdong China; 5 Department of Sports Medicine, Peking University Shenzhen Hospital, Shenzhen Peking University-The Hong Kong University of Science and Technology Medical Center, Shenzhen, 518036 China; 6 Department of Oncology, Zhujiang Hospital, Southern Medical University, Guangzhou, 510282 Guangdong China; 7 Menzies Institute for Medical Research, University of Tasmania, Hobart, 7001 Australia; 8 Clinical Research Centre, Tsinghua Medicine, Beijing Tsinghua Changgung Hospital, Tsinghua University, Beijing, 102218 China; 9 Women’s Hospital of Nanjing Medical University, Nanjing Women and Children’ s Healthcare Hospital, Nanjing, 210004 China

**Keywords:** DNA hydrogel, Osteoarthritis, Early diagnosis, Therapy

## Abstract

Osteoarthritis (OA) is a chronic inflammatory disease characterized primarily by cartilage degradation. Current imaging techniques often detect OA only after irreversible cartilage damage has occurred, and no drugs are available to halt disease progression. Early diagnosis and intervention may serve as an effective strategy to delay OA advancement. In this study, we developed a multifunctional material TEMPO@DSH by encapsulating 2,2,6,6-tetramethylpiperidine 1-oxyl (TEMPO), a reactive oxygen species (ROS)-responsive antioxidant, within anti-inflammatory DNA supramolecular hydrogel (DSH). TEMPO@DSH mitigates oxidative stress-induced mitochondrial damage in chondrocytes and inhibits the NF-κB and mTOR pathways, thereby reducing chondrocyte senescence and exerting therapeutic effects against OA. Additionally, by taking advantage of the loss of paramagnetism of TEMPO after reacting with ROS, magnetic resonance imaging following intra-articular injection of TEMPO@DSH can reflect the local levels of ROS in the joint. Given that elevated ROS levels precede observable imaging changes in early-stage OA, this approach enables early diagnosis. Overall, TEMPO@DSH serves as an integrated platform for the early diagnosis and treatment of OA, potentially offering a novel strategy for effective disease management.

**Graphical abstract:**

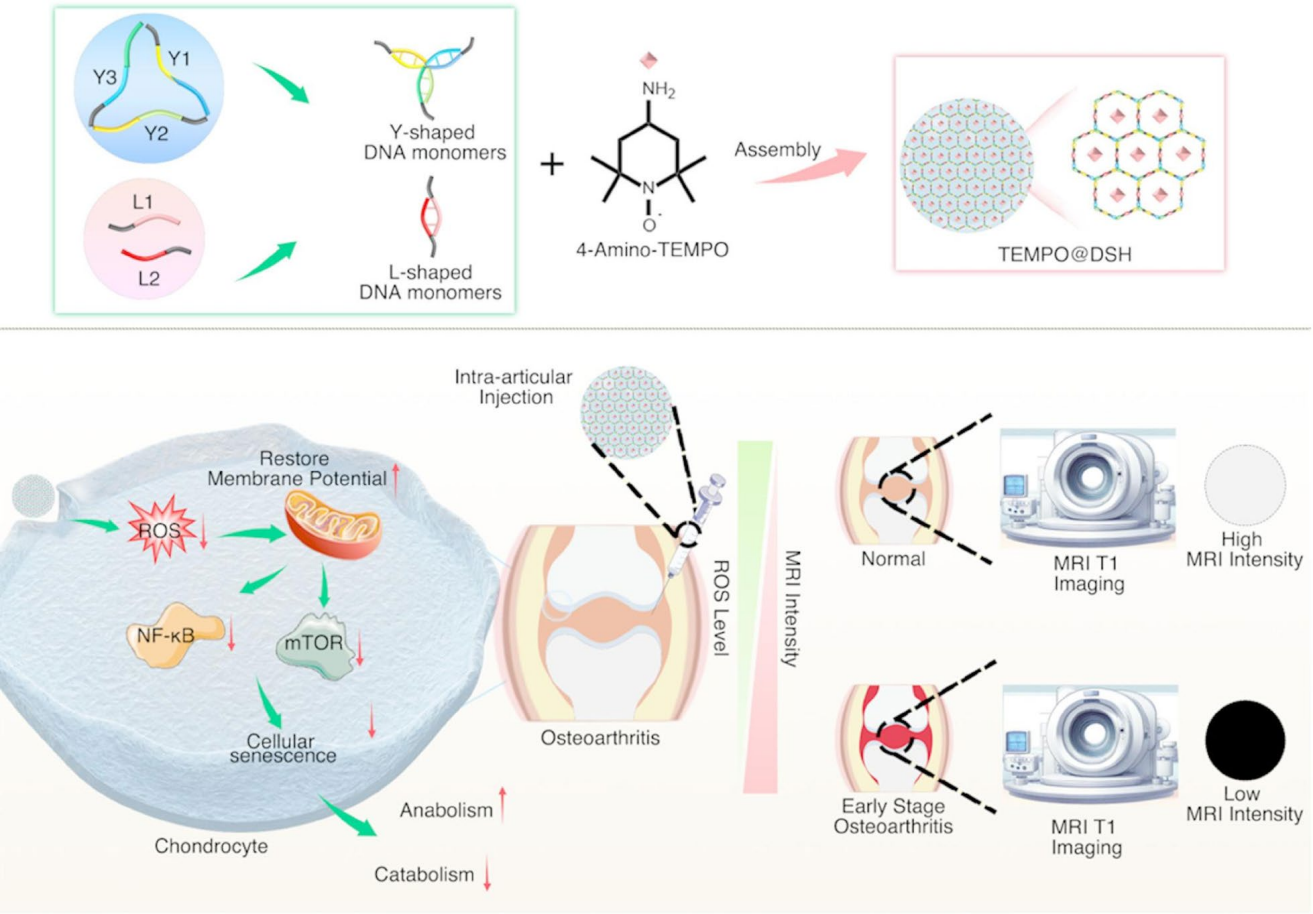

**Supplementary Information:**

The online version contains supplementary material available at 10.1186/s12951-025-03794-0.

## Introduction

Osteoarthritis (OA), a prevalent musculoskeletal disorder most frequently affecting the knee joint, stands as a leading cause of disability among the elderly population [1, 2]. With the growing aging demographics and increasing obesity rates, its incidence is projected to rise substantially [3]. Pathological changes in OA affect the entire joint, primarily characterized by cartilage destruction [4]. However, current imaging modalities typically detect OA only after irreversible cartilage damage has occurred, thereby missing critical opportunities for early intervention [5, 6]. Chronic inflammation and oxidative stress play pivotal roles in OA progression by inducing mitochondrial dysfunction, disrupting chondrocyte homeostasis, and accelerating cellular senescence and apoptosis, ultimately driving joint degeneration [7–9]. To date, no pharmacological agents have been approved to halt or reverse OA progression. Targeting initial pathological changes through early detection and intervention could provide an effective approach to slowing OA progression [6].

DNA supramolecular hydrogels (DSH) are three-dimensional network-structured drug carriers formed through the base-pairing assembly of single-stranded DNA (ssDNA), enabling the physical encapsulation of therapeutic agents within the hydrogel matrix [10]. DSH exhibits low toxicity, high biocompatibility, and minimal immunogenicity, making them promising candidates for drug carriers [10, 11]. Furthermore, the abilities of DSH to scavenge ROS and inflammatory factors endow it with therapeutic potential for treating inflammation-related diseases, making it an ideal carrier for localized treatment of inflammatory diseases [12–14].

2,2,6,6-Tetramethylpiperidine-1-oxyl (TEMPO), a piperidine-derived nitroxide radical, exhibits antioxidant effects through scavenging peroxyl radicals [15, 16]. Given that oxidative stress constitutes a key pathological hallmark of OA and that the mitigation of ROS levels effectively retards disease progression [17], intra-articular TEMPO administration may therefore confer chondroprotective effects against OA. Additionally, the paramagnetic nature of the nitroxide group in TEMPO allows it to influence the magnetic resonance imaging (MRI) signals of surrounding water molecules, thereby enhancing image contrast [18, 19]. Upon reacting with peroxyl radicals to form 2,2,6,6-tetramethylpiperidine-1-hydroxy (TEMPO-H), TEMPO loses its paramagnetic properties; this characteristic enables indirect quantification of local oxidative stress levels via MRI [18]. Compared to healthy joints, OA joints generate excessive peroxyl radicals even at early stages. Intra-articular TEMPO administration could thus enable early OA diagnosis through MRI-based detection of elevated oxidative stress.

**Fig. 1:**
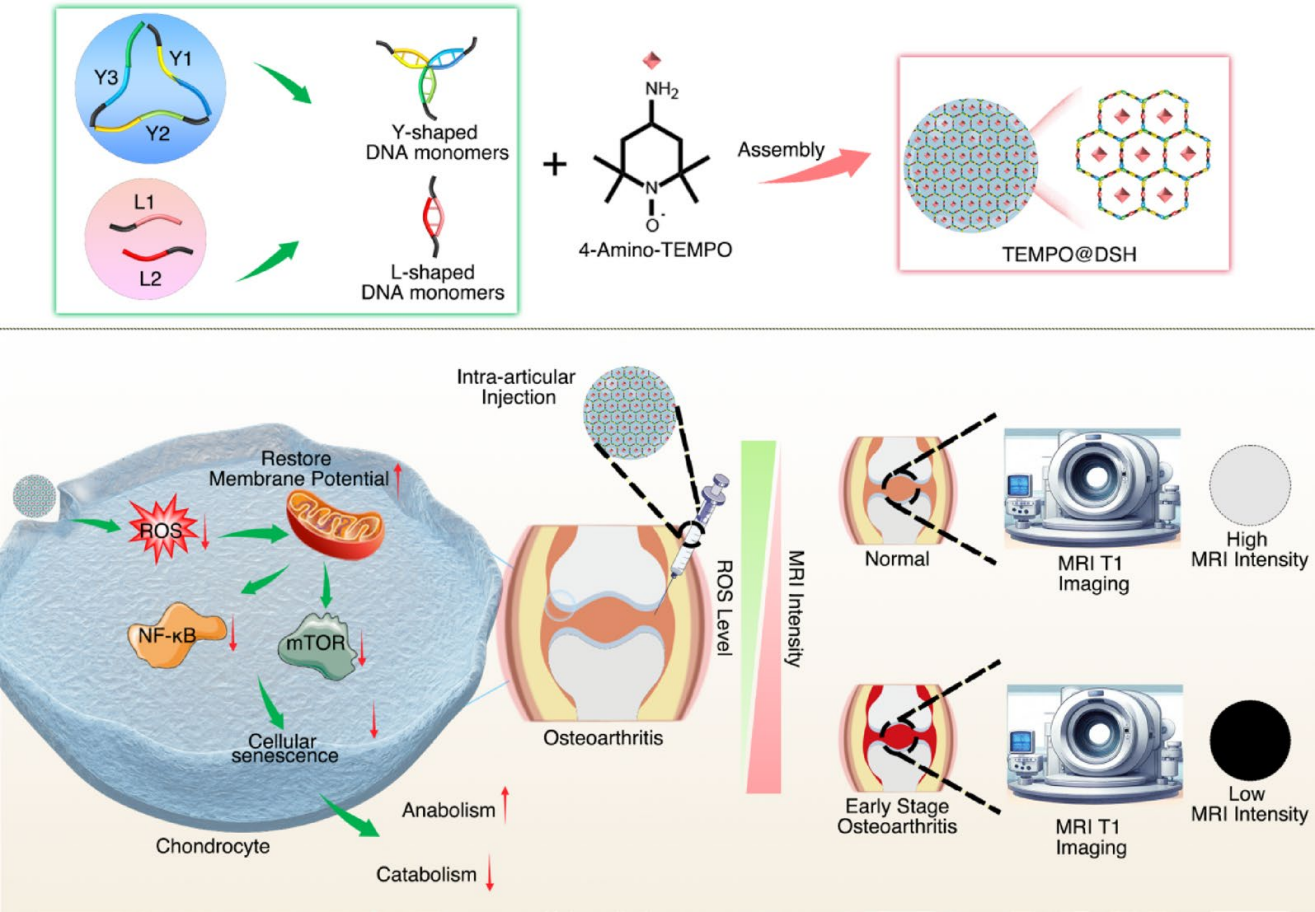
Preparation and working principle of TEMPO@DSH. Upper panel: Schematic diagram of TEMPO@DSH synthesis. Lower left panel: Schematic illustration of the potential therapeutic mechanism of TEMPO@DSH in osteoarthritis treatment. Lower right panel: Schematic representation of TEMPO@DSH application for early osteoarthritis diagnosis

In this study, we synthesized a novel TEMPO-loaded DSH (TEMPO@DSH) by integrating TEMPO with Y-scaffold (Y) and linear linker (L) DNA monomers. Our findings indicate that TEMPO@DSH holds promise as an MRI contrast agent for the early detection of OA. Furthermore, intra-articular administration of TEMPO@DSH reduces ROS generation and mitigates mitochondrial damage in OA chondrocytes, thereby delaying cellular senescence and exerting therapeutic effects against OA. Thus, this innovative material offers an integrated approach for the early diagnosis and treatment of OA, presenting a promising strategy for timely disease intervention (Fig. 1).​.

## Materials and methods

### Preparation of DSH and TEMPO@DSH

To synthesize TEMPO@DSH, partially complementary single-stranded DNA sequences (Supplementary Table 1) were designed for preparing Y- and L- DNA monomers. For Y-monomers, strands Y1, Y2, and Y3 (12.5 µL, 2 mM for each strand) were mixed with 62.5 µL PBS (pH 7.2, 2 mM MgCl₂), denatured at 95 °C for 5 min, and annealed by cooling to 4 °C. Similarly, L1 and L2 strands (18.75 µL, 2 mM for each strand) were combined with 62.5 µL PBS (pH 7.2, 2 mM MgCl₂) and processed identically to form L-monomers.

For DSH assembly, Y-monomers (32 µL, 250 µM) and L-monomers (32 µL, 375 µM) were mixed with 36 µL PBS (pH 7.2, 2 mM MgCl₂), triggering rapid gelation (< 5 s). TEMPO@DSH was prepared by premixing TEMPO (36 µL, 25 µg/mL) with Y- and L-monomers under identical conditions, achieving gelation within 2 min at room temperature alongside a sol-to-gel transition.

### Characterization of DSH and TEMPO@DSH

The morphology and microstructure of DSH were characterized by scanning electron microscopy (Magellan 400). The molecular weights of single-stranded DNA (Y1, Y2, Y3, L1, L2) and the Y/L-monomers were analyzed using polyacrylamide gel electrophoresis. Oscillatory time sweep tests were performed on DSH and TEMPO@DSH using an ARES rheometer (TA Instruments, USA). Samples were carefully loaded between parallel plates (25 mm diameter, 1 mm gap) and allowed to rest for 5 min to minimize shear history effects. Measurements were conducted at 37℃ under a constant frequency of 1 Hz (6.28 rad/s) and a strain amplitude of 1% (within the linear viscoelastic region). The storage modulus (G′) and loss modulus (G″) were monitored over time. To evaluate the encapsulation efficiency of TEMPO in DSH, NHS-Cy5.5-labeled TEMPO was mixed with the hydrogel, and fluorescence imaging was performed using an inverted microscope (Nikon) to validate successful encapsulation. Oscillatory strain sweep tests on TEMPO@DSH were conducted using an ARES rheometer (TA Instruments, USA). Samples were loaded between parallel plates (25 mm diameter, 1 mm gap) and allowed to rest for 5 min after loading. Measurements were performed at 25℃ with a fixed frequency of 1 Hz (≈ 6.28 rad/s) and a strain range of 0.1%–1000%. The storage modulus (G′) and loss modulus (G″) were measured as functions of strain to determine the linear viscoelastic region and the yield point (yield strain) of the material. To verify DSH encapsulation of TEMPO, TEMPO was first conjugated with NHS-Cy5.5 in 1× PBS (pH 7.8) for 4 h (10:1 molar ratio), followed by 24-hour dialysis (MWCO 500 Da) to remove excess TEMPO. After mixing the NHS-Cy5.5-labelled TEMPO with the DNA single strands to form hydrogels, images were captured using an inverted fluorescence microscope (Nikon, Germany). The drug loading capacity of TEMPO in TEMPO@DSH was calculated as (weight of TEMPO/weight of TEMPO@DSH) × 100%.

### Electron paramagnetic resonance (EPR) experiments

The signal of Cy5.5, TEMPO, and the TEMPO-Cy5.5 was tested by EPR. Briefly, 100 mL of Cy5.5 (168 µg/ml), TEMPO (25 µg/ml), and the TEMPO-Cy5.5 (193 µg/ml) suspension were transferred into sealed glass capillary with 0.9e1.1 mm diameter and then the glass capillary was inserted into the resonant cavity. EPR detection was carried out and the spectrum was recorded at room temperature by using a Bruker EMXplus-10/12 spectrometer (Bruker, Germany) operating at 9.85 GHz with a variable temperature control unit (Bruker ER4141 VT-I).

### TEMPO release study

To evaluate the release of TEMPO (Cy5.5-labeled) from the TEMPO@DSH, the hydrogel was placed in a dialysis bag (molecular weight cutoff: 3.5–14 kDa) and incubated at 37℃ in the dark (to prevent Cy5.5 hydrolysis) using either synovial fluid or medium containing 10% fetal bovine serum (FBS). A control group was set up with PBS (without hydrogel) in the dialysis bag. The dialysis bag was immersed in 10 mL of PBS buffer (pH 7.4). At predetermined time points (0, 0.5, 1, 2, 4, 8, 12, 24, and 48 h), 100 µL of the release medium was withdrawn from outside the dialysis bag and replaced with an equal volume of fresh PBS. The amount of TEMPO released was quantified using a fluorescence microplate reader (Cy5.5, excitation/emission wavelengths: 675/695 nm), and the percentage of drug cumulative release at each time point was calculated as (Cumulative release amount of TEMPO released up to time point *t*/Initial TEMPO content) × 100%.

### Chondrocyte isolation and culture‌

Knee cartilage tissues were obtained from OA patients undergoing knee arthroplasty at the Department of Orthopaedics, Zhujiang Hospital, Southern Medical University. This study was approved by the Institutional Review Board of Zhujiang Hospital (Approval No. 2019KY02203), with written informed consent obtained from all participants before tissue collection.

Primary chondrocytes were isolated through sequential enzymatic digestion. Briefly, cartilage tissues were rinsed three times with PBS containing 10% penicillin-streptomycin (100 U/mL and 100 µg/mL, respectively; Sigma-Aldrich), then minced into 0.5 mm³ fragments using sterile scalpels. The minced cartilage was digested with 0.25% trypsin (0.02% EDTA) for 30 min at 37 °C. Subsequently, the tissue fragments were digested with 0.2% type II collagenase for 16–24 h on a constant temperature shaker (37 °C, 90 rpm). Isolated chondrocytes were seeded at a density of 1 × 10⁴ cells/cm² in culture flasks containing DMEM/F12 medium supplemented with 10% fetal bovine serum and 1% penicillin-streptomycin (Sigma-Aldrich). Cells were maintained at 37 °C in a 5% CO₂ atmosphere, with medium changes every 48 h. Subculture was performed using 0.25% trypsin (0.02% EDTA) when cells reached 80% confluence.

### Analysis of cellular uptake

NHS-Cy5-labelled TEMPO and TEMPO@DSH were added to chondrocytes, which were subsequently incubated for 24 h at 37 °C under 5% CO_2_. After incubation, the cytoskeleton was labelled with β-actin and the nucleus was stained with DAPI. Cellular uptake was imaged using an inverted fluorescence microscope (Nikon).

### Cell viability assay

To assess the drug’s effect on cell viability, a CCK-8 assay was performed. Human primary chondrocytes were seeded at a density of 5 × 10³ cells/well in 96-well plates and allowed to adhere overnight. After 24 h of drug exposure, the medium was aspirated, and each well was gently washed with PBS. Subsequently, 100 µL of fresh medium containing 10% CCK-8 reagent (Beyotime Biotechnology) was added, followed by incubation at 37 °C under 5% CO₂ for 2 h. Absorbance was measured at 450 nm using a microplate reader (BioTek) to assess cell viability.

### Cytotoxicity assay

Chondrocytes were incubated with 500 µL of calcein-AM/propidium iodide cytodye (AM/PI) for 15 min. In viable cells, calcein-AM is hydrolyzed by intracellular esterases to calcein, which emits bright green fluorescence. Conversely, PI selectively enters dead cells through compromised membranes and intercalates into nuclear DNA, emitting red fluorescence. Fluorescence images were captured using a confocal microscope (Leica) to assess cell viability and death.

### Treatment of chondrocytes

Chondrocytes were seeded in six-well plates at a density of 1 × 10⁵ cells/well and divided into five experimental groups: (1) PBS (control), (2) IL-1β, (3) IL-1β + TEMPO, (4) IL-1β + DSH, and (5) IL-1β + TEMPO@DSH. After 24 h of pretreatment with PBS, PBS, TEMPO (25 µg/mL), DSH (2.5 mM), and TEMPO@DSH (containing 25 µg/mL TEMPO and 2.5 mM DSH) for the five groups respectively, cells in all groups except the control group were stimulated with 10 ng/mL IL-1β for an additional 24 h.

### Quantification of H₂O₂

To evaluate the ability of TEMPO, DSH, and TEMPO@DSH to scavenge ROS in a cell-free system, H₂O₂ was added to PBS, TEMPO, DSH, and TEMPO@DSH solutions, respectively. After the reaction, the remaining H₂O₂ concentration was measured using a hydrogen peroxide assay kit (Solarbio, BC3595). Briefly, 100 µL of H₂O₂ (250 mM) was added to 100 µL of each of the following solutions: PBS, TEMPO (9 µg/mL), DSH (50 mM), and TEMPO@DSH (containing 9 µg/mL TEMPO and 50 mM DSH). The assay was performed following the manufacturer’s instructions. The remaining H₂O₂ in each group was then quantified using a spectrophotometer at a wavelength of 415 nm.

### Measurement of intracellular ROS

The culture medium was aspirated from each well, and chondrocytes were gently washed three times with sterile PBS. Cells were incubated with 10 µM DCFH-DA working solution for 20 min at 37 °C in the dark. After incubation, cells were washed twice with PBS to remove the excess probe, and 2 mL of serum-free medium was added to terminate the reaction. Fluorescence images were captured using an inverted fluorescence microscope (Nikon), and fluorescence intensity was quantified with ImageJ software.

### Analysis of the mitochondrial membrane potential

An appropriate amount of JC-1 (200×) was prepared, and 50 µL of JC-1 was diluted with 8 mL of ultrapure water. The solution was stirred to ensure complete dissolution and mixing. The mixture was then diluted with JC-1 staining buffer (5×) to prepare the JC-1 staining working solution. The chondrocytes were washed with PBS, after which the working solution was added and incubated for 30 min at 37 °C. After washing, the chondrocytes were cultured in a serum-free medium, and images were acquired using an inverted fluorescence microscope (Nikon). The percentage of mitochondrial depolarization was determined by measuring the relative red-to-green fluorescence ratio.

### Quantitative real-time polymerase chain reaction (qRT-PCR) analysis

Total RNA was isolated using a TRIzol reagent. Reverse transcription of purified RNA was performed with the High-Capacity cDNA Reverse Transcription Kit. mRNA expression levels were quantified using SYBR Premix Ex Taq Master Mix (2×) (Takara), and relative target gene expression was calculated by the comparative Ct (ΔΔCt) method. Validated primer sequences for each gene are listed in Table S2. Data normalization was achieved using glyceraldehyde-3-phosphate dehydrogenase (GAPDH) as an internal control.

### Western blot analysis

Chondrocytes were washed with ice-cold PBS. After removing excess PBS from the six-well plates, the cells were lysed by adding radioimmunoprecipitation assay (RIPA) buffer (containing 1% phenylmethylsulfonyl fluoride) to each well. The collected proteins were quantified and denatured by boiling at 100 °C. Denatured proteins were separated by sodium dodecyl sulfate-polyacrylamide gel electrophoresis (SDS-PAGE) and transferred to a polyvinylidene fluoride (PVDF) membrane. The PVDF membrane was blocked with 5% BSA. The membrane was incubated with the following primary antibodies at 4 °C for 12 h: ACAN (1:2000; Abcam, ab315486), COL2A1 (1:2000; Abcam, ab34712), SOX9 (1:1000; Proteintech, Cat# 67439-1-lg), MMP13 (1:2000; Abcam, ab39012), MMP3 (1:2000; Abcam, ab52915), ADAMTS5 (1:2000; Abcam, ab41037), p-mTORC (1:2000; Abcam, ab109268), mTOR (1:2000; Abcam, ab134903), p-S6K (1:2000; Cell Signaling Technology, 4858 S), S6K (1:3000; Santa Cruz Biotechnology, sc-74459), NFκB1 (1:2000; Proteintech, 14220-1-AP), p-p65 (1:1000; Proteintech, 82335-1-RR), p65 (1:1000; Proteintech, 10745-1-AP), GAPDH (1:5000; Proteintech, 60004-1-lg). The membrane was then incubated with an HRP-conjugated secondary antibody at room temperature for 2 h. Immunoreactivity was detected using ultrasensitive chemiluminescent reagents. The intensity of each protein band was normalized to glyceraldehyde-3-phosphate dehydrogenase (GAPDH) or total protein (for phosphorylated proteins).

### Alcian blue and toluidine blue staining

Chondrocytes were seeded into 12-well plates and treated with designated drugs. After drug intervention, cells were cultured for 14 days with medium replenishment every three days. Following fixation in 4% paraformaldehyde and three PBS washes, proteoglycan was assessed by Toluidine blue or Alcian blue staining for 15 min (Solarbio). Excess dyes were removed by gentle rinsing with distilled water, and stained matrices were visualized under a light microscope (Nikon).

### Cartilage explants

Cartilage was isolated from fresh human knee joint specimens, cut into small pieces (5 mm × 5 mm × 2 mm), and then treated with the corresponding drugs according to experimental groups. The explants were maintained in DMEM (Gibco) supplemented with 10% fetal bovine serum (Invitrogen) and 1% penicillin-streptomycin (Sigma-Aldrich). After a 10-day incubation period, the explants were collected for histological examination.

### SA-β-Gal staining

Chondrocytes were fixed with SA-β-gal staining fixative (Beyotime) at room temperature for 15 min, followed by three washes with PBS. Cells were then incubated in SA-β-gal staining solution at 37 °C for 12 h. SA-β-gal-positive cells were visualized and imaged under an inverted light microscope (Leica).

### Immunofluorescence staining

Chondrocytes were seeded into confocal dishes. After treatment, cells were washed three times with sterile PBS and fixed with 4% paraformaldehyde. Following permeabilization with 0.1% Triton X-100, samples were blocked with 5% bovine serum albumin (MedChemExpress) for 1 h at room temperature. The primary antibody against γ-H2AX (1:200; Abcam, ab11175) was diluted and applied to completely cover the samples, and incubated on a shaker at 4 °C for 12 h. After washing with PBS, a fluorescent secondary antibody was diluted and applied to the samples, and incubated at room temperature for 1 h in the dark. Following PBS washes, nuclei were counterstained with 4′,6-diamidino-2-phenylindole (DAPI) contained in an anti-fade mounting medium. Coverslips were carefully placed face-down onto the mounting medium to seal the samples. Fluorescence signals were visualized using an inverted fluorescence microscope (Nikon), and images were analyzed with ImageJ software.

### RNA-Seq analysis

After treating OA-modeled chondrocytes with TEMPO@DSH and PBS, total RNA was extracted using TRIzol reagent (Takara Bio). The samples were subjected to next-generation sequencing on an Illumina platform by Lianchuan Biotechnology Co., Ltd. Each transcriptomic analysis utilized human chondrocyte samples containing 2× 10⁵ cells. Kyoto Encyclopedia of Genes and Genomes (KEGG) pathway analysis and Gene Set Enrichment Analysis (GSEA) were performed to identify pathways associated with therapeutic targets in OA chondrocytes.

### OA model

All experiments were approved by the Animal Care and Use Committee of Zhujiang Hospital, Southern Medical University (LAEC-2022-122). Ten-week-old male C57BL/6J mice and Sprague-Dawley (SD) rats were used to establish OA models. Experimental OA was induced by surgical destabilization of the medial meniscus (DMM) in the right knee joint, while sham-operated controls received joint capsule incision without ligament disruption. After successful modeling, mice were randomly assigned to four treatment groups: PBS group, TEMPO group, DSH group, and TEMPO@DSH group. Each group received a 6 µL intra-articular injection of their respective treatment: PBS, TEMPO (500 µg/ml), DSH (50 mM), or TEMPO@DSH (containing 500 µg/mL TEMPO and 50 mM DSH). Injections were administered slowly to ensure even distribution within the joint cavity. Eight weeks post-surgery, mice were euthanized, and knee joint specimens were harvested for histological evaluation.

### MRI imaging of TEMPO@DSH

Magnetic resonance imaging (MRI) examinations were performed using a 7.0 T preclinical MRI system (PharmScan 70/16 US; Bruker BioSpin MRI GmbH; 200-mm horizontal bore). To investigate whether TEMPO@DSH can serve as an indicator of ROS levels under MRI, TEMPO@DSH (containing 500 µg/mL TEMPO and 50 mM DSH) were mixed with H₂O₂ solutions at different concentrations (0, 100, 200, and 500 nM) and incubated at 37℃ in the dark for 5 min to simulate an oxidative stress environment. Subsequently, T1-weighted imaging (T1WI) was acquired with the following parameters: repetition time (TR) = 500 ms, echo time (TE) = 10 ms, slice thickness = 2 mm, matrix size = 256 × 256, and field of view (FOV) = 200 mm × 200 mm. The T1-weighted signal intensity of each reaction system was quantified using ImageJ software. In the animal imaging experiments, rats were anesthetized with isoflurane, and 50 µL of TEMPO@DSH (containing 500 µg/mL TEMPO and 50 mM DSH) was intra-articularly injected into the knee joint. Then, T1WI sequences were acquired with the following parameters: echo time (TE) = 7.31 ms, repetition time (TR) = 350 ms, slice thickness = 0.7 mm, and field of view (FOV) = 25.6 mm. The acquired imageswere processed and pseudocolored using Paravision software, and regions of interest were quantified using ImageJ.

### Retention of TEMPO in knee joints

Both TEMPO and TEMPO@DSH were covalently conjugated to NHS-Cy5.5 for fluorescent labeling. Equivalent molar doses of the labeled compounds (6 µL of TEMPO at a concentration of 500 µg/mL) were administered via intra-articular injection into the mice knee joints. Post-injection, in vivo imaging was performed at predetermined time points using a Multispectral Imaging System (PerkinElmer) to monitor the distribution and retention of the labeled compounds within the joint cavity.

### Stability of TEMPO@DSH in synovial fluid

TEMPO@DSH was incubated with the synovial fluid for 0, 1, 2, 3, 5, 9, and 14 days. Subsequently, the mixture was diluted with an equal volume of PBS, and 5× DNA loading buffer was added. Samples from each group were analyzed by agarose gel electrophoresis to assess TEMPO@DSH degradation.

### Histological assessment

Eight weeks post-surgery, the mice were euthanized. The muscle and fascia of the knee joint specimens were removed, and the samples were fixed in 4% paraformaldehyde for one day. Decalcification was then performed using a 4% EDTA decalcification solution for two weeks. Following fixation and decalcification, the samples were embedded in paraffin blocks and sectioned at predetermined anatomical locations for further histological analysis. The paraffin-embedded tissue sections were subjected to histological staining using Safranin O/Fast Green (Solarbio), Toluidine Blue (Solarbio), and Hematoxylin-Eosin (Sigma-Aldrich) to evaluate cartilage morphology, proteoglycan content, and overall tissue structure, respectively. For Safranin O/Fast Green staining, paraffin-embedded tissue sections were sequentially stained with 0.2% Safranin O solution for 3 min to visualize proteoglycans, followed by 0.2% Fast Green solution for 1 min to counterstain collagenous matrix. For Toluidine Blue staining, sections were incubated with Toluidine Blue for 1 min to assess metachromatic proteoglycan distribution. For Hematoxylin-Eosin staining, sections were first stained with Hematoxylin for 3 min to highlight nuclei, rinsed with PBS to remove excess dye, and then counterstained with Eosin to delineate cytoplasmic and extracellular matrix components. Histological evaluation was conducted based on the Osteoarthritis Research Society International (OARSI) scoring system and the Krenn synovitis score.

### Immunohistochemistry assay

The sections were baked at 68 °C, followed by immersion in xylene and a graded alcohol series for dewaxing and rehydration. Subsequently, antigen retrieval was performed by incubating the sections in diluted sodium citrate-EDTA buffer at 62 °C in a water bath for 12 h. Endogenous peroxidase activity was quenched using 3% H₂O₂, and the sections were blocked with immunohistochemical blocking buffer for 1 h. MMP-13 antibody (1:200; Abcam, ab39012), COL2A1 antibody (1:200; Abcam, ab34712), p-mTORC (1:2000; Abcam, ab109268), p-S6K (1: 2,000, Cell Signaling Technology, 4858 s), p-p65 antibody (1:200; proteintech, 82335-1-RR), NFκB1 antibody (1:2000; proteintech, 14220-1-AP), p16 INK4a antibody (1:200; proteintech, 10883-1-AP) and p21 antibody (1:200; proteintech, 10355-1-AP) were used to incubate the sectioned tissues at 4℃ for 12 h. After PBS washes, a horseradish peroxidase (HRP)-conjugated secondary antibody (1:200; ABclonal, AS014) was applied at room temperature for 1 h. Images were acquired using an inverted light microscope (Leica).

### Statistical analysis

Statistical analyses were performed using GraphPad Prism 8.0.2. Error bars in the figures represent standard deviation or 95% confidence intervals, as detailed in the legends. Comparisons between groups were performed using either an unpaired two-tailed Student’s t-test or the Mann-Whitney U test, depending on data distribution. Statistical significance was defined as *P* < 0.05.

## Results and discussion

### Synthesis and characterization of TEMPO@DSH

**Fig. 2:**
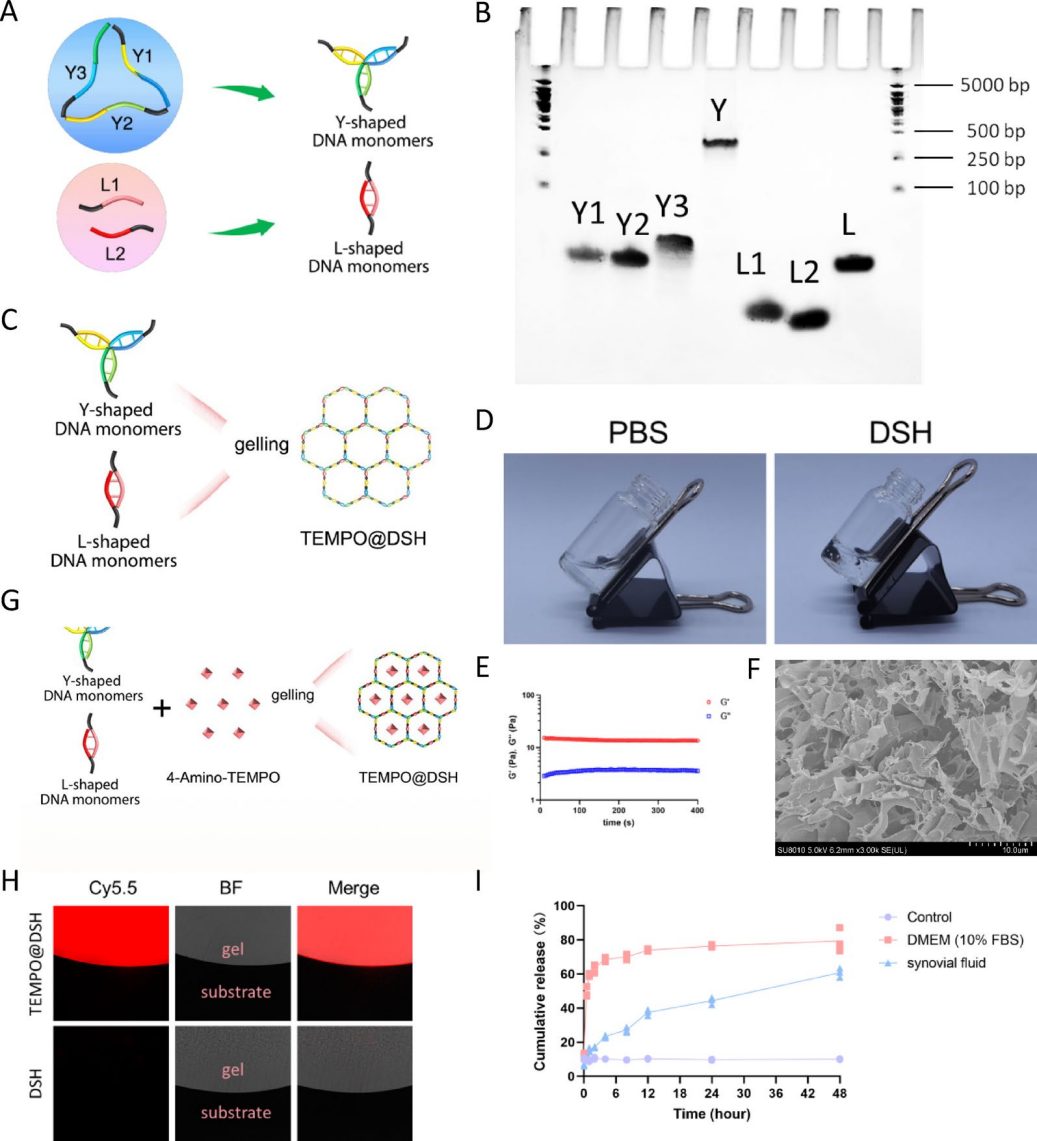
Synthesis and characterization of DSH and TEMPO@DSH. (**A**) Schematic diagram illustrating the synthesis of Y and L DNA monomers. (**B**) Polyacrylamide gel electrophoresis characterization of ssDNA and Y and L DNA monomers. (**C**) Schematic diagram illustrating the synthesis of DSH. (**D**) Photographs showing the gel state of DSH. (**E**) Rheology analysis of DSH. The red line indicated storage modulus (G’) and the blue line indicated loss modulus (G’’). (**F**) Scanning electron microscope image of DSH. (**G**) Schematic diagram illustrating the synthesis of TEMPO@DSH. (**H**) Fluorescence images of TEMPO@DSH. TEMPO labeled with Cy5.5 was encapsulated within DSH. (**I**) Cumulative release of TEMPO from TEMPO@DSH in DMEM (10% FBS) and synovial fluid over 48 h

To synthesize DSH, Y and L DNA monomers were prepared using ssDNAs with partially complementary base pairing (Fig. 2A, Table S1). Polyacrylamide gel electrophoresis revealed that the migration rates of Y-monomers and L-monomers were slower than those of their complementary ssDNAs, and the molecular weights of all ssDNAs and DNA monomers matched our design, confirming the successful formation of Y- and L-monomers (Fig. 2B).

Upon simply mixing the Y- and L-monomers at room temperature, DSH was immediately assembled, with the mixture transitioning from a solution to a gel state within 5 s (Fig. 2C and D). Oscillatory shear stress of rheological analysis demonstrated that the shear loss modulus (G″) of the synthesized DSH was approximately 15 times lower than its shear storage modulus (G′), exhibiting distinct viscoelastic behavior that indicates DSH possesses typical hydrogel characteristics (Fig. 2E). Scanning electron microscopy revealed that DSH exhibits a three-dimensional porous microstructure, suggesting its excellent potential for drug loading and release, which is considered a critical advantage of DSH (Fig. 2F). Additionally, DSH, composed of natural DNA molecules, exhibits excellent biocompatibility and biodegradability, thus presenting lower risks of systemic toxicity and immune responses [20–22]. Simultaneously, DSH demonstrates favorable injectability, facilitating minimally invasive administration and in vivo molding, making it suitable for localized treatments within complex tissue environments [14, 23]. Taken together, the unique advantages of DSH in drug delivery make it an ideal carrier for the treatment of OA.

TEMPO@DSH was formed by mixing TEMPO with Y- and L-monomers (Fig. 2G). To evaluate whether TEMPO was uniformly encapsulated within DSH, NHS-Cy5.5-labeled TEMPO was used to synthesize TEMPO@DSH. Observation of EPR signals in TEMPO and TEMPO-Cy5.5, but not in Cy5.5 (Figure S1), confirmed the successful conjugation of paramagnetic TEMPO with Cy5.5. As shown in Fig. 2H, the Cy5.5 fluorescence signal was evenly distributed and overlapped with the DSH matrix, demonstrating homogeneous encapsulation of TEMPO within the hydrogel. We calculated the drug loading capacity of TEMPO@DSH as (weight of TEMPO/weight of TEMPO@DSH) × 100%, which was 0.145%. The rheological time sweep experiment revealed that the storage modulus (G’) was significantly higher than the loss modulus (G’’) (Figure S2A), indicating that TEMPO@DSH exhibited predominantly elastic behavior. A strain sweep experiment was conducted to assess the viscoelastic properties of TEMPO@DSH, and the observed yielding transition at high strain further supports its favorable injectability (Figure S2B). We also investigated the release behavior of TEMPO from TEMPO@DSH. The results showed that, compared with its rapid release in DMEM (10% FBS), TEMPO exhibited a slower release in synovial fluid, with approximately 60% cumulative release after 48 h (Fig. 2I). The release profile of TEMPO in synovial fluid suggests that loading with DSH may confer a sustained-release property to TEMPO within the joint cavity.

### TEMPO@DSH reduces ROS and maintains anabolic-catabolic homeostasis in chondrocytes

**Fig. 3:**
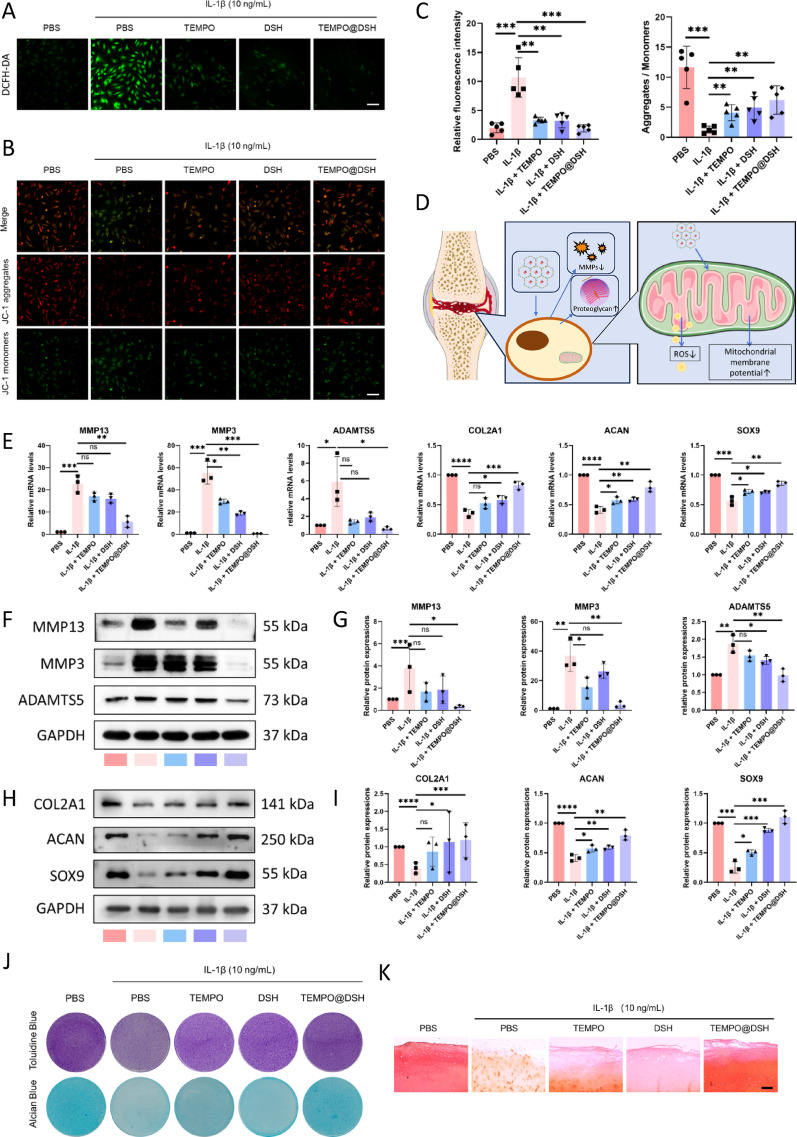
TEMPO@DSH exerts a protective effect on osteoarthritic chondrocytes. (**A**) Measurement of ROS in chondrocytes following the indicated treatments. (**B**) Detection of mitochondrial membrane potential (relative fluorescence intensity of red/green) in chondrocytes following the indicated treatments. (**C**) Quantification data of Fig. 3A and B (*n* = 3). (**D**) Schematic diagram of the antioxidant effects of TEMPO@DSH on chondrocytes. (**E**) qRT-PCR analysis for the mRNA levels of MMP13, MMP3, ADAMTS5, COL2A1, ACAN, and SOX9 in chondrocytes following the indicated treatments. (**F**) Western-blot analysis for protein levels of MMP13, MMP3, and ADAMTS5 in chondrocytes following the indicated treatments. (**G**) Quantification data of Fig. 3F (*n* = 3). (**H**) Western-blot analysis for protein levels of COL2A1, ACAN, and SOX9 in chondrocytes following the indicated treatments. (**I**) Quantification data of Fig. 3H (*n* = 3). (**J**) Toluidine blue and Alcian blue staining of chondrocytes following the indicated treatments. (**K**) Safranin O staining of human cartilage explants following the indicated treatments. Scale bar: 60 μm. For C, E, G, and I data are represented as means ± standard deviation. *P < 0.05. ***P < 0.001. ****P < 0.0001

In this study, we found that TEMPO could still be taken up by chondrocytes after being encapsulated in DSH (Figure S3). At appropriate concentrations, TEMPO@DSH treatment showed no significant cytotoxicity and even increased cell viability in chondrocytes (Figures S4−7). This increase in cell viability may be attributed to TEMPO’s ROS scavenging activity, as well as DSH’s anti-inflammatory effects and its ability to mimic the extracellular matrix environment. ROS-induced oxidative stress, a key characteristic of osteoarthritic chondrocytes, can trigger mitochondrial damage [24]. Mitochondrial damage results in decreased ATP production, increased ROS generation, and impaired mitophagy, which further disrupts chondrocyte homeostasis, forming a vicious cycle that promotes OA progression [25]. In the cell-free system, TEMPO@DSH effectively reduced H₂O₂ levels, demonstrating its capacity for ROS scavenging (Figure S8). To investigate the antioxidant and mitochondrial protective effects of TEMPO@DSH in chondrocytes, we measured cellular ROS levels and mitochondrial membrane potential. The results showed that both TEMPO and DSH alone, to some extent, inhibited the production of ROS and the decrease in mitochondrial membrane potential in OA-modeled chondrocytes, while TEMPO@DSH treatment exerted the most pronounced inhibitory effect on oxidative stress and mitochondrial damage (Fig. 3A and C). These findings suggest that TEMPO@DSH can effectively mitigate ROS accumulation and mitochondrial dysfunction in chondrocytes (Fig. 3D).

**Fig. 4:**
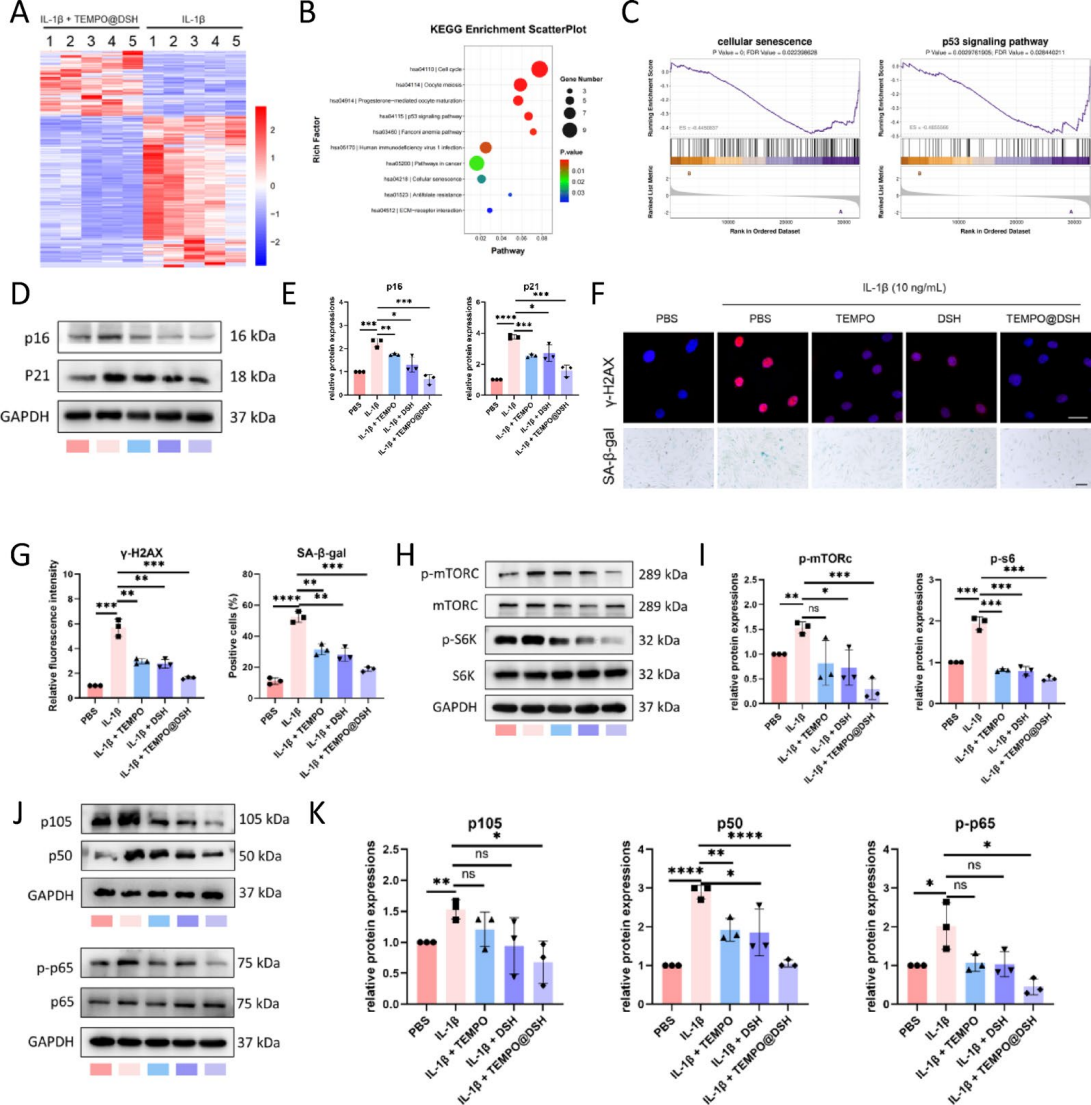
DSH@TEMPO delays senescence and its related pathways in chondrocytes. (**A**) Heatmap of differentially expressed genes between DSH@TEMPO treated chondrocytes and untreated OA-modelled chondrocytes. (**B**) KEGG pathway enrichment analysis of the differentially expressed genes identified in Fig. 3A. (**C**) GSEA for cellular senescence and p53 signaling pathways. (**D**) Western blot analysis for the protein levels of p16 and p21 in chondrocytes following the indicated treatments. (**E**) Quantification data of Fig. 4D (*n* = 3). (**F**) γ-H2AX immunofluorescent staining and SA-β-gal staining of chondrocytes following the indicated treatments. (**G**) Quantifying the fluorescence intensity of γ-H2AX and the positive cells for SA-β-gal in Fig. 4F (*n* = 3). (**H**) Western blot analysis for the protein levels of p-mTORC, mTORC, p-S6K, and S6K in chondrocytes following the indicated treatments. (**I**) Quantification data of Fig. 4H (*n* = 3). (**J**) Western blot analysis for the protein levels of p105, p50, p-p65, and p65 in chondrocytes following the indicated treatments. (**K**) Quantification data of Fig. 4J (*n* = 3). For E, G, I, and K, data are represented as means ± standard deviation. *P < 0.05, **P < 0.01, ***P < 0.001, and ****P < 0.0001

**Fig. 5:**
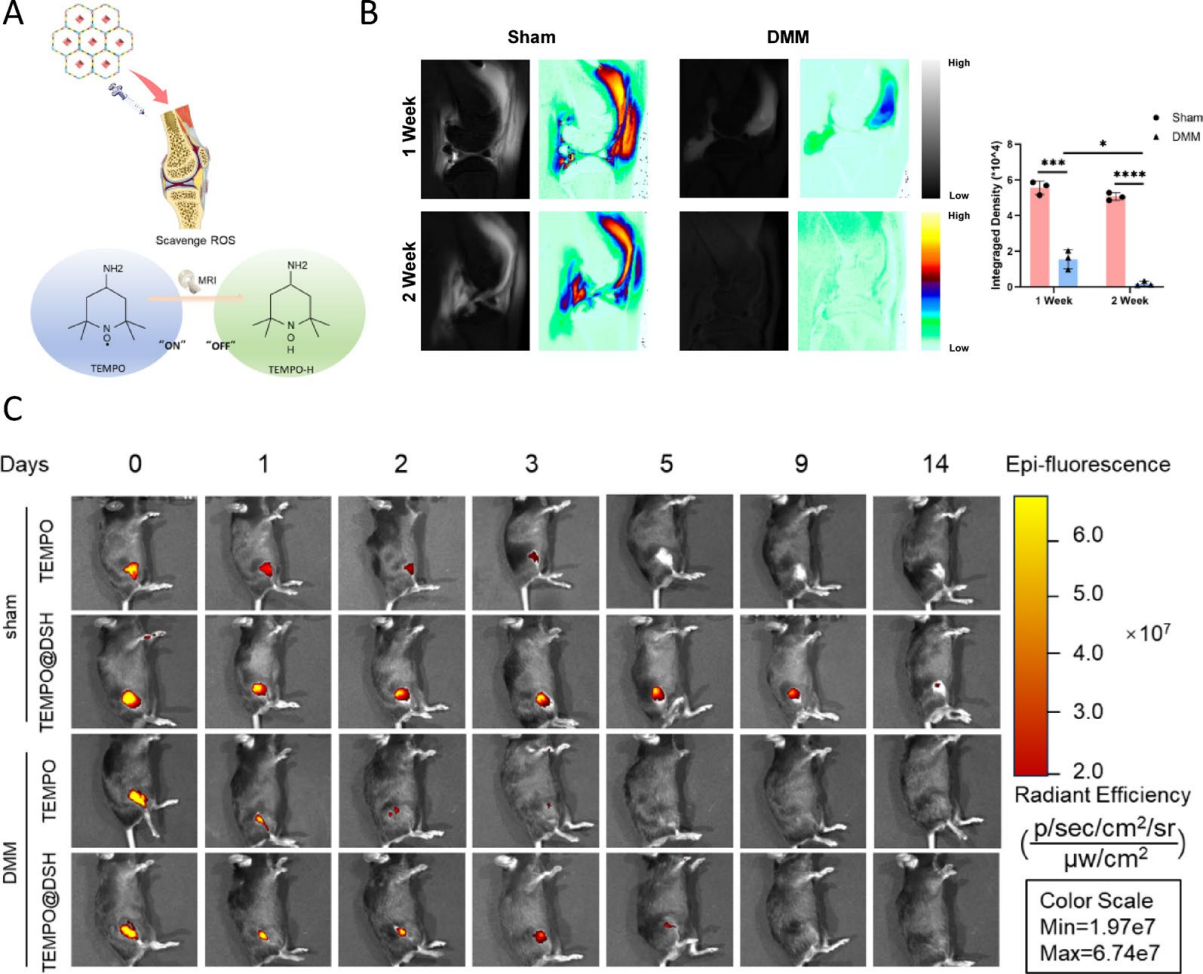
MRI and in vivo tracking of TEMPO@DSH following intra-knee injection. (**A**) Schematic diagram of the mechanism of TEMPO@DSH as a MRI contrast agent for early diagnosis of knee osteoarthritis. (**B**) T1-weighted images of the joint cavity using TEMPO@DSH as the contrast agent. (**C**) In vivo imaging of TEMPO and TEMPO@DSH-treated mice at specified time points. For (**B**), data are represented as means ± standard deviation, and a two-tailed Student’s t-test was used for comparison, *P < 0.05

**Fig. 6:**
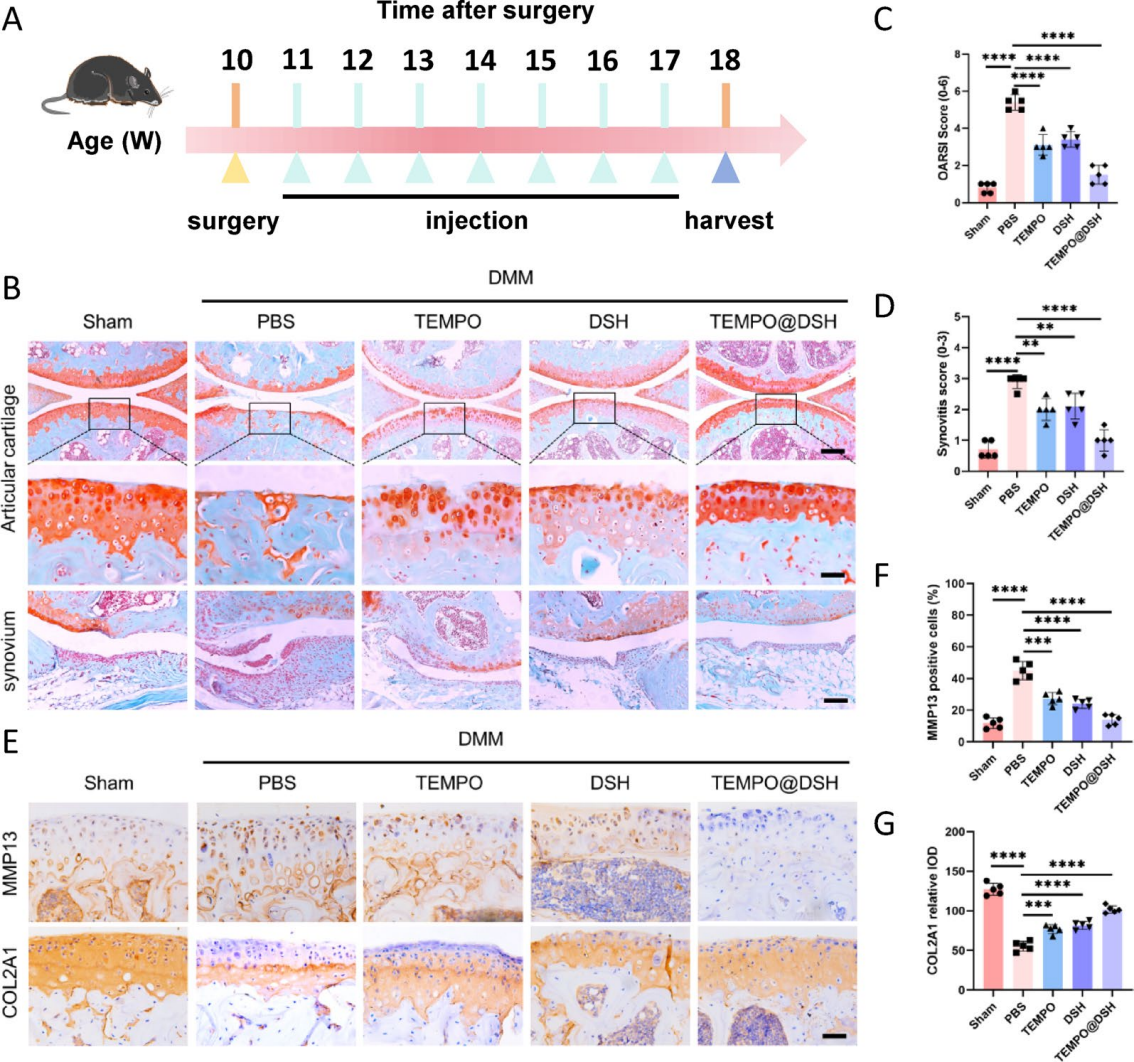
Therapeutic effect of TEMPO@DSH on osteoarthritis model mice. (**A**) Experimental scheme of the drug administration in osteoarthritis mice. (**B**) Safranin O/fast green staining of mice knee joints following the indicated treatments. Top panel: low-magnification image of cartilage; dashed boxes indicate regions magnified below. Scale bar: 60 μm. Middle panel: the high-magnification image of the top panel. Scale bar: 60 μm. Bottom panel: image of synovium. Scale bar: 100 μm. (**C**) OARSI score of mice knee joints following the indicated treatments (*n* = 5). (**D**) Synovitis score of mice knee joints following the indicated treatments (*n* = 5). (**E**) Immunohistochemistry staining of MMP13 and COL2A1 in articular cartilage following the indicated treatments. Scale bars: 60 μm. (**F**) Quantifying the positive cells for MMP13 in Fig. 6E (*n* = 5). (**G**) Quantifying the relative Integral Optical Density (IOD) of COL2A1 in Fig. 6E (*n* = 5). For (**C**) and (**D**), data are present as mean values ± 95% confidence interval, and a Mann-Whitney U test was used for comparison. For (**F**) and (**G**), data are represented as means ± standard deviation, and two-tailed Student’s t test was used for comparison. **P < 0.01, ***P < 0.001, and ****P < 0.0001

**Fig. 7:**
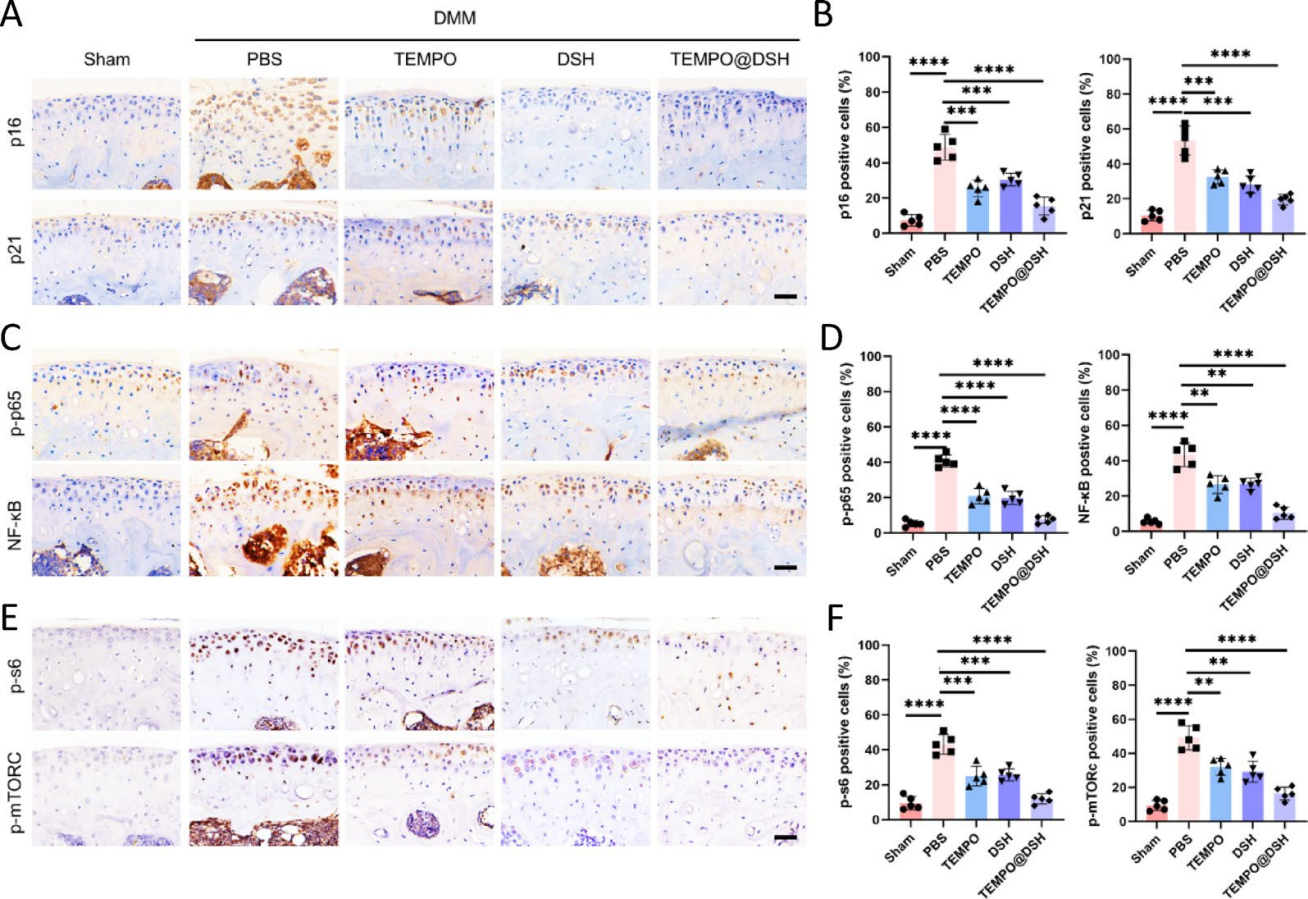
TEMPO@DSH inhibits chondrocyte senescence and senescence-related pathways in osteoarthritis model mice. (**A**) Immunohistochemistry staining of p16 and p21 in articular cartilage following the indicated treatments. Scale bars: 60 μm. (**B**) Quantifying the positive cells for p16 and p21 in Fig. 7A (*n* = 5). (**C**) Immunohistochemistry staining of p-p65 and NF-κB in articular cartilage following the indicated treatments. Scale bars: 60 μm. (**D**) Quantifying the positive cells for p-p65 and NF-κB in Fig. 7C (*n* = 5). (**E**) Immunohistochemistry staining of p-s6 and p-mTORC in articular cartilage following the indicated treatments. Scale bars: 60 μm. (**F**) Quantifying the positive cells for p-s6 and p-mTORC in Fig. 7E (*n* = 5). For (**B**), (**D**), and (**G**), data are represented as means ± standard deviation, and a two-tailed Student’s t-test was used for comparison. **P < 0.01, ***P < 0.001, and ****P < 0.0001

Mitochondrial dysfunction promotes a shift from anabolic to catabolic metabolism in chondrocyte [26]. The balance between catabolic and anabolic metabolism is crucial for maintaining joint health, and its disruption can accelerate chondrocyte extracellular matrix (ECM) degradation, contributing to OA pathogenesis [27]. Therefore, we further evaluated the effects of TEMPO@DSH on both catabolic and anabolic metabolism as well as the ECM in chondrocytes. Experimental data revealed that TEMPO@DSH treatment significantly downregulated the expression of catabolic markers (MMP-13, MMP-3, and ADAMTS5) and upregulated anabolic markers (COL2A1, Aggrecan, and SOX9) in OA-modeled chondrocytes, with superior efficacy compared with TEMPO or DSH alone (Fig. 3E and I). Additionally, ECM staining demonstrated that TEMPO@DSH markedly enhanced glycosaminoglycan levels in both chondrocytes and human cartilage explants (Fig. 3J and K). Collectively, these results indicate that TEMPO@DSH may protect OA chondrocytes by alleviating oxidative stress-induced mitochondrial damage, thereby restoring metabolic equilibrium and ECM homeostasis.

### TEMPO@DSH inhibits senescence and senescence-related pathways in chondrocytes

To elucidate the mechanism underlying the chondroprotective effects of TEMPO@DSH, we performed transcriptomic sequencing on IL-1β-induced OA-modeled chondrocytes treated with or without TEMPO@DSH (Fig. 4A, Figure S9). Gene enrichment analysis revealed significant enrichment of senescence-related pathways between the two groups (Fig. 4B and C, S10). Senescence is a key feature of OA chondrocytes, and mitochondrial dysfunction can drive chondrocyte senescence [28, 29]. To validate whether TEMPO@DSH alleviates OA chondrocyte senescence, we further examined senescence-related markers. Results demonstrated that IL-1β-induced chondrocytes exhibited markedly increased senescence; treatment with either TEMPO or DSH partially reduced senescence, whereas TEMPO@DSH exerted the most pronounced inhibitory effect on chondrocyte senescence (Fig. 4D and G).

Studies suggest that mitochondrial damage activates the mTOR and NF-κB signaling pathways, which play pivotal roles in chondrocyte senescence [30–33]. To explore the potential mechanism by which TEMPO@DSH exerts its anti-senescence effects, we examined the expression of these pathways. Both TEMPO and DSH suppressed the IL-1β-induced upregulation of mTOR and NF-κB pathways, with TEMPO@DSH showing the strongest inhibitory effect (Fig. 4H and K). These findings suggest that TEMPO@DSH may mitigate chondrocyte senescence by suppressing the activation of the mTOR and NF-κB signaling pathways.

### TEMPO@DSH serves as a contrast agent for early diagnosis of knee OA

TEMPO contains nitroxide radicals (·NO) that confer paramagnetic properties, enabling it to influence the magnetic resonance signals of surrounding water molecules and thereby alter image contrast [19]. When TEMPO reacts with ROS, it is converted into TEMPO-H and loses its paramagnetic character [19]. By reacting with different concentrations of H₂O₂, TEMPO@DSH was demonstrated to serve as an indicator of ROS levels under MRI (Figure S11). In early-stage knee OA, excessive ROS is generated even before obvious cartilage damage occurs. Consequently, TEMPO injected into an osteoarthritic knee may react with ROS and become depleted, resulting in a weaker MRI signal compared to that from TEMPO injected into a healthy knee. Therefore, this mechanism could provide a potential approach for early OA diagnosis through MRI (Fig. 5A).

To validate this hypothesis, TEMPO@DSH was intra-articularly injected into rats with early-stage OA and sham-operated controls, followed by T1-weighted MRI. The imaging results revealed that the MRI signal intensity in the joint cavity of early-stage OA rats was significantly lower than that in the sham-operated group. Moreover, OA-modeled mice at 2 weeks post-induction exhibited significantly reduced MRI signal intensity compared to those at 1 week (Fig. 5B). These findings suggest that TEMPO@DSH can serve as an MRI-detectable contrast agent reflecting local ROS levels in joints, highlighting its potential for early OA diagnosis.

### TEMPO@DSH exhibits a protective effect against OA

DSH, serving as a drug carrier, forms a densely crosslinked three-dimensional network via covalent bonding, which restricts drug molecules to diffuse gradually through the network pores [34, 35]. Moreover, the hydrogen bonding interactions between DSH and the encapsulated drugs further impede their free diffusion, thereby conferring DSH with sustained drug release properties [36, 37]. To evaluate whether DSH can extend the retention time of TEMPO within the joint cavity, we performed in vivo imaging to monitor the retention of TEMPO and TEMPO@DSH in the mouse knee joint. The results demonstrated that DSH can, to a certain extent, prolong the retention time of TEMPO in the joint cavity (Fig. 5C). In addition, we assessed the stability of TEMPO@DSH in human synovial fluid and found that TEMPO@DSH could still be detected 14 days after incubation (Figure S12), indicating its excellent stability in synovial fluid.

To assess the therapeutic potential of TEMPO@DSH for OA, we administered weekly intra-articular injections to mice one week after OA modeling (Fig. 6A). At 8 weeks post-modeling, we observed that treatment with TEMPO or DSH alone partially alleviated cartilage damage, catabolic-anabolic imbalance, and synovial inflammation (Fig. 6B-D, S13, S14). However, TEMPO@DSH demonstrated superior OA-protective effects compared to TEMPO or DSH alone (Fig. 6B-D, S9, S10). Furthermore, we investigated the potential visceral toxicity of TEMPO@DSH and found no significant morphological abnormalities in the major organs of treated mice (Figure S15). Collectively, these findings suggest that TEMPO@DSH possesses both effective and safe therapeutic potential for the treatment of knee OA.

### TEMPO@DSH suppresses chondrocyte senescence and senescence-related pathways in vivo‌

To further verify whether TEMPO@DSH exerts OA-protective effects by suppressing chondrocyte senescence, we examined the expression of senescence marker proteins in the knee cartilage of the mice. The results demonstrated that both TEMPO and DSH could partially reduce OA model-induced chondrocyte senescence, while TEMPO@DSH exhibited a more pronounced reduction in senescent chondrocytes (Fig. 7A-B). Additionally, we investigated the expression of senescence-related signaling pathways in vivo and found that TEMPO@DSH more effectively suppressed the activation of NF-κB and mTOR signaling pathways in OA chondrocytes compared to TEMPO or DSH alone (Fig. 7C-F). These findings suggest that TEMPO@DSH may exert protective effects against OA by delaying chondrocyte senescence through inhibition of NF-κB and mTOR pathways.

## Conclusion

In summary, we developed a multifunctional material, TEMPO@DSH, by encapsulating TEMPO within DSH. This material enables MRI-based early diagnosis of OA by reacting with ROS in the joint. Furthermore, TEMPO@DSH may delay chondrocyte senescence and thereby treat OA by reducing oxidative stress-induced mitochondrial damage and inhibiting the NF-κB and mTOR pathways. Therefore, our designed TEMPO@DSH offers a promising, integrated strategy for the early diagnosis and intervention of OA, representing an ideal approach for the prevention and treatment of this disease.

## Supplementary Information

Supplementary Material 1.

## Data Availability

No datasets were generated or analysed during the current study.
