# Supplementary material for: A TEMPO-loaded DNA hydrogel enabling integrated early diagnosis and treatment of osteoarthritis
Source: J Nanobiotechnology. 2025 Oct 31;23:695. doi: 10.1186/s12951-025-03794-0 (PMC12577105; doi:10.1186/s12951-025-03794-0)
Supplement: Supplementary file 1 — Supplementary Material 1. [file 12951_2025_3794_MOESM1_ESM.docx]

**A TEMPO-loaded DNA Hydrogel Enabling Integrated Early Diagnosis and Treatment of Osteoarthritis**

*Hong Huang^1, 2, 3^†, Mingze Tang^4^†, Pengcheng Hu^3^†, Yingshi Zhan^3^†, Wei Sun^1, 2^, Weipeng Zheng^1^, Jianwei Zhu^1^, Jianmao Chen^3^, Song Xue^5^, Shiqian Huang^3^, Weiyu Han^4^, Chao Zhang^6^, Changhai Ding^1, 2, 3, 7, 8^*, Yan Zhang^3^*, Shushu Li^9^*, Guangfeng Ruan^1, 2^**

1. Department of Orthopedics, Guangzhou First People's Hospital, School of Medicine, South China University of Technology, Guangzhou, Guangdong, 510180, China
2. Clinical Research Centre, Guangzhou First People's Hospital, Guangzhou Medical University, Guangzhou, Guangdong, 510180, China
3. Clinical Research Center, Zhujiang Hospital, Southern Medical University, Guangzhou, Guangdong, 510282, China
4. Centre of Orthopedics, Zhujiang Hospital, Southern Medical University, Guangzhou, Guangdong, 510282, China
5. Department of Sports Medicine, Peking University Shenzhen Hospital, Shenzhen Peking University-The Hong Kong University of Science and Technology Medical Center, Shenzhen, 518036, China
6. Department of Oncology, Zhujiang Hospital, Southern Medical University, Guangzhou, Guangdong, 510282, China
7. Menzies Institute for Medical Research, University of Tasmania, Hobart 7001, Australia
8. Clinical Research Centre, Beijing Tsinghua Changgung Hospital, Tsinghua Medicine, Tsinghua University, Beijing, 102218, China
9. Women's Hospital of Nanjing Medical University, Nanjing Women and Children' s Healthcare Hospital, Nanjing 210004, China.

† These authors contributed equally.

*Corresponding authors: Changhai Ding (changhai.ding@utas.edu.au); Yan Zhang ([yan.zhang@utas.edu.au);](mailto:weiyu.han@utas.edu.au;) Shushu Li ([lishushu@njmu.edu.cn](mailto:lishushu@njmu.edu.cn)[);](mailto:czhangsinap@163.com;) Guangfeng Ruan ([ruan1989.ok@163.com](mailto:ruan1989.ok@163.com)).

Supplementary Materials for


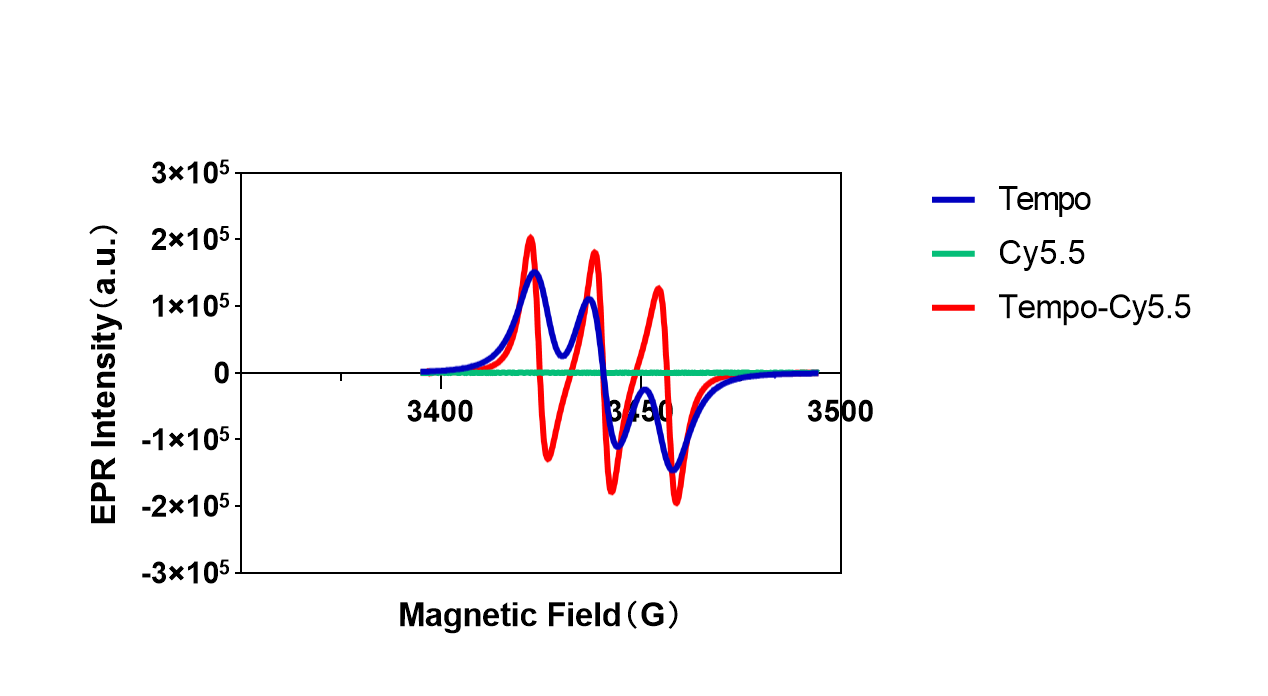


**Figure S1.** Electron Paramagnetic Resonance spectra of Cy5.5, TEMPO and TEMPO-Cy5.5.


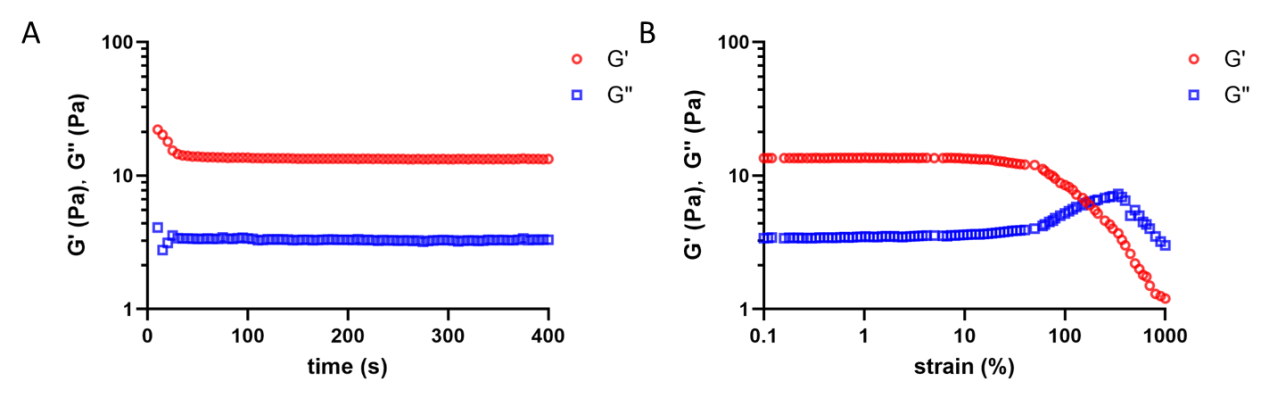


**Figure S2.** Rheology analysis of TEMPO@DSH. The red line indicated storage modulus (G') and the blue line indicated loss modulus (G''). (A) Time sweep test performed at 37℃ with a strain of 1% and a frequency of 1 Hz. (B) Strain sweep test performed at 25℃ with a fixed frequency of 1 Hz (≈6.28 rad/s) and a strain range of 0.1%–1000%.

**Figure S3.** Cellular uptake of TEMPO following 24-hour treatment with Cy5.5-labeled TEMPO and TEMPO@DSH. Nuclei are stained with DAPI (blue). Cytoskeletons are visualized by β-actin immunofluorescence staining (green). Scale bars: 50 µm.

**Figure S4.** CCK8-detected chondrocyte viability following 24-hour treatment with TEMPO at the indicated concentration.

**Figure S5.** CCK8-detected chondrocyte viability following 24-hour treatment with DSH at the indicated concentration.

**Figure S6.** CCK8-detected chondrocyte viability following 24-hour treatment with TEMPO@DSH at the indicated concentration.

**Figure S7.** Acetoxymethyl ester /Propidium Iodide (AM/PI)-based assessment of chondrocyte cytotoxicity following 24-hour exposure to specified treatments. AM is used to label live cells (green fluorescence), and PI is used to label dead cells (red fluorescence). Scale bars: 100 µm.


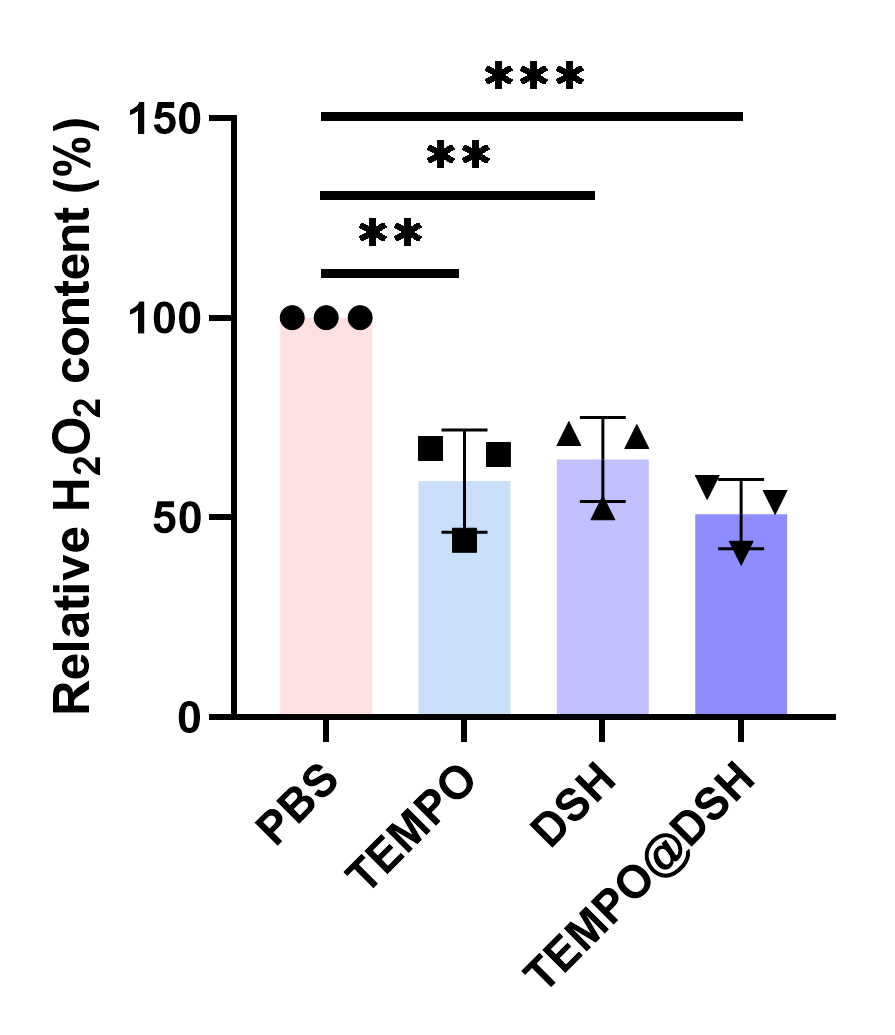


**Figure S8. Effects of different materials on the content of H₂O₂ at the same concentration.** Each group was treated with 250 mM H₂O₂ solution, and the remaining H₂O₂ levels were measured after the reaction. Data are represented as means ± standard deviation. One-way analysis of variance (ANOVA) was used to compare the differences between groups. ***P* < 0.01, ****P* < 0.001.


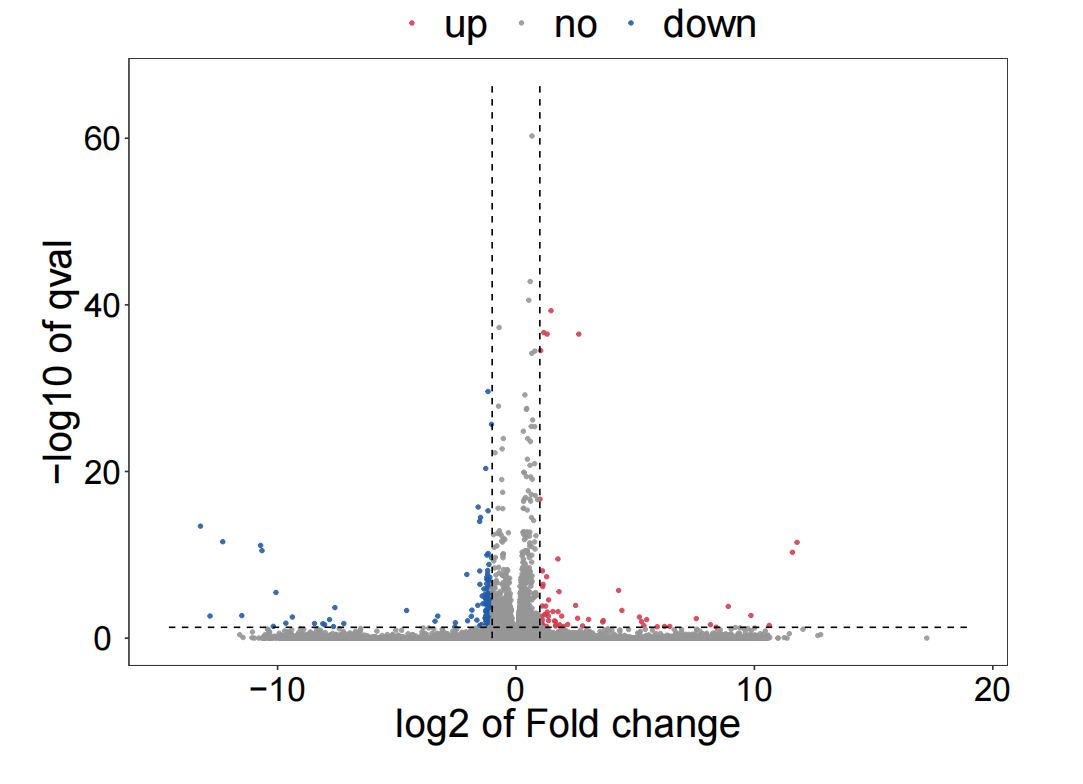


**Figure S9.** Volcano plot of differentially expressed genes from transcriptome sequencing of osteoarthritis-modeled chondrocytes with and without TEMPO@DSH treatment.


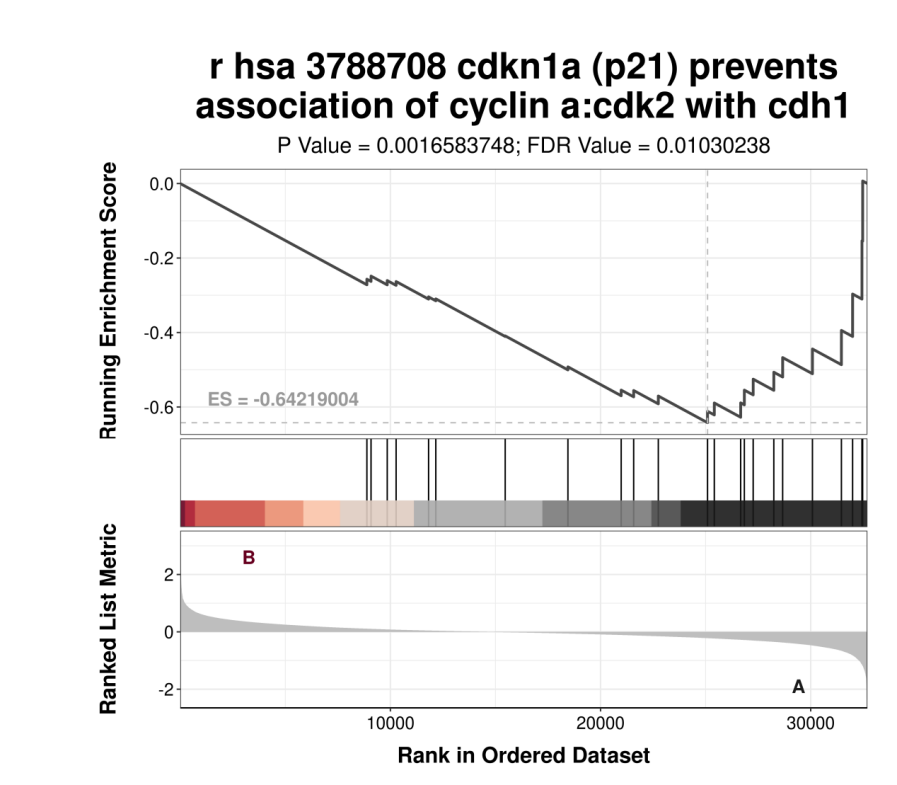


**Figure S10.** Gene Set Enrichment Analysis for p21-related pathways.

**Figure S11. MRI images of TEMPO@DSH after reaction with H₂O₂.** (A) T1-weighted magnetic resonance imaging (MRI) of TEMPO@DSH after reaction with different concentrations of H2O2. (B) Quantitative data of MRI signal intensity from Figure S11A (n=3). Data are represented as means ± standard deviation. One-way analysis of variance (ANOVA) was used to compare the differences between groups. ***P* < 0.01, *****P* < 0.0001, ns: non-significant.

**Figure S12.** **Degradation Analysis of TEMPO@DSH in Synovial Fluid.** (A) Agarose gel electrophoresis of TEMPO@DSH in synovial fluid at the indicated time points. (B) Quantification data of Figure S12A (n=3).

**Figure S13.** Toluidine staining of the cartilage following the indicated treatments. Scale bar: 60 μm.

**Figure S14.** Hematoxylin and Eosin staining of the cartilage following the indicated treatments. Scale bar: 60 μm.

**Figure S15.** Histological morphologies of major organs in mice following the indicated treatments. Scale bar: 100 μm.

**Supplementary Tables S1. Detailed sequences of Y1, Y2, Y3, L1, L2.**

| **Name** | **DNA sequence (5’-3’)** |
| --- | --- |
| Y1 | TATAGTCTAGAGCCGGAGCATGAAAAGATTGGGATATAGTATAATCATCAT |
| Y2 | TATAGTCTAGAATGATGATTATACTATATCCCACCTGACTCCTGGGAGAAG |
| Y3 | TATAGTCTAGACTTCTCCCAGGAGTCAGGTGCAATCTTTTCATGCTCCGGC |
| L1 | TCTAGACTATAACTAGATACATACAG |
| L2 | TCTAGACTATACTGTATGTATCTAGT |

**Supplementary Tables S2. Human Primers used for qRT-PCR.**

| **Gene** | **Forward primer (5’-3’)** | **Reverse primer (5’-3’)** |
| --- | --- | --- |
| GAPDH | CTGGGCTACTACTGAGCACC | AAGTGGTCGTTGAGGGCAATG |
| MMP-13 | GACTTCCCAGGAATTGGTGA | TGACGCGAACAATACGGTTA |
| MMP-3 | CGGTTCCGCCTGTCTCAAG | CGCCAAAAGTGCCTGTCTT |
| ADAMTS5 | TTGTTCCACCTCCTCGCATC | GCTTTATCCTGGGCAGGTGT |
| COL2A1 | TGGACGCCATGAAGGTTTTCT | TGGGAGCCAGATTGTCATCTC |
| ACAN | GTGCCTATCAGGACAAGGTCT | GATGCCTTTCACCACGACTTC |
| SOX9 | AGCGAACGCACATCAAGAC | CTGTAGGCGATCTGTTGGGG |

GAPDH: glyceraldehyde-3-phosphate dehydrogenase; MMP13: matrix metallopeptidase 13; MMP3: matrix metallopeptidase 3; ADAMTS5: A Disintegrin And Metalloproteinase with Thrombospondin Motifs 5; COL2A1: collagen type II alpha 1 chain; ACAN: aggrecan; SOX9: SRY-box transcription factor 9.
